# DCA-UNet: A Cross-Modal Ginkgo Crown Recognition Method Based on Multi-Source Data

**DOI:** 10.3390/plants15020249

**Published:** 2026-01-13

**Authors:** Yunzhi Guo, Yang Yu, Yan Li, Mengyuan Chen, Wenwen Kong, Yunpeng Zhao, Fei Liu

**Affiliations:** 1 College of Mathematics and Computer Science, Zhejiang A&F University, Hangzhou 311300, China; 2023611011009@stu.zafu.edu.cn (Y.G.);; 2 State Key Laboratory for Vegetation Structure, Function and Construction (VegLab), College of Biosystems Engineering and Food Science, Zhejiang University, Hangzhou 310058, China; 3 Zhejiang Provincial Animal Husbandry Technology Extension and Breeding Livestock and Poultry Monitoring Station, Hangzhou 310020, China; 4 MOE Key Laboratory of Biosystem Homeostasis and Protection, College of Life Sciences, Zhejiang University, Hangzhou 310058, China; ypzhao@zju.edu.cn

**Keywords:** RGB, multispectral, UAV, ginkgo crown, semantic segmentation, deep learning, attention mechanism

## Abstract

Wild ginkgo, as an endangered species, holds significant value for genetic resource conservation, yet its practical applications face numerous challenges. Traditional field surveys are inefficient in mountainous mixed forests, while satellite remote sensing is limited by spatial resolution. Current deep learning approaches relying on single-source data or merely simple multi-source fusion fail to fully exploit information, leading to suboptimal recognition performance. This study presents a multimodal ginkgo crown dataset, comprising RGB and multispectral images acquired by an UAV platform. To achieve precise crown segmentation with this data, we propose a novel dual-branch dynamic weighting fusion network, termed dual-branch cross-modal attention-enhanced UNet (DCA-UNet). We design a dual-branch encoder (DBE) with a two-stream architecture for independent feature extraction from each modality. We further develop a cross-modal interaction fusion module (CIF), employing cross-modal attention and learnable dynamic weights to boost multi-source information fusion. Additionally, we introduce an attention-enhanced decoder (AED) that combines progressive upsampling with a hybrid channel-spatial attention mechanism, thereby effectively utilizing multi-scale features and enhancing boundary semantic consistency. Evaluation on the ginkgo dataset demonstrates that DCA-UNet achieves a segmentation performance of 93.42% IoU (Intersection over Union), 96.82% PA (Pixel Accuracy), 96.38% Precision, and 96.60% F1-score. These results outperform differential feature attention fusion network (DFAFNet) by 12.19%, 6.37%, 4.62%, and 6.95%, respectively, and surpasses the single-modality baselines (RGB or multispectral) in all metrics. Superior performance on cross-flight-altitude data further validates the model’s strong generalization capability and robustness in complex scenarios. These results demonstrate the superiority of DCA-UNet in UAV-based multimodal ginkgo crown recognition, offering a reliable and efficient solution for monitoring wild endangered tree species.

## 1. Introduction

Ginkgo (*Ginkgo biloba* L.), the world’s oldest surviving relict plant, possesses remarkable medicinal, timber, ornamental, and scientific value. However, its wild populations currently face severe threats from genetic erosion and habitat contraction. Due to insufficient systematic conservation and excessive exploitation, wild ginkgo has been classified as an endangered species [1]. Conducting germplasm resource surveys is therefore of great theoretical and practical significance for establishing gene banks, breeding superior cultivars, and promoting ecological restoration [2,3,4].

Wild ginkgo mainly occurs on semi-shaded, high-humidity slopes at elevations of 500–1000 m, typically coexisting with coniferous-broadleaved mixed forests that exhibit complex stand structures and severe canopy occlusion [5,6,7,8]. Traditional field surveys in such terrains are labor-intensive, time-consuming, and highly susceptible to observer bias. In contrast, satellite remote sensing provides wide coverage and short revisit cycles, offering a feasible solution for regional-scale forest monitoring [9,10]. Previous studies have demonstrated its application potential. For instance, Andresini achieved semantic segmentation of forest mortality areas by fusing Sentinel-1 and Sentinel-2 data, confirming the critical role of satellite imagery in forest health monitoring [11]. Lin successfully identified individual olive trees using high-resolution DigitalGlobe imagery combined with semantic segmentation and vision transformers. Further studies have significantly improved satellite-based tree species classification [12]. Gazzea developed a weakly supervised framework on 0.5 m WorldView-2 imagery, raising the mean F1-score for spruce, pine, and broadleaf classes from 0.33 to 0.85 [13]. Qin employed Zhuhai-1 hyperspectral data and random forest to perform pixel-level classification of 28 dominant urban tree species, achieving an overall accuracy of 76.8% [14]. Bu proposed robust small object detection YOLO(OD-YOLO), which enhanced small-target detection and multi-scale feature fusion based on YOLOv8n, improving mAP50 by 5.2% on the VisDrone dataset [15]. Despite these advances, satellite remote sensing remains constrained by relatively low spatial resolution and susceptibility to atmospheric interference, limiting its capability for precise individual tree crown delineation in densely occluded mixed forests [16,17].

UAV remote sensing, with its ultra-high spatial resolution, flexibility, and relatively low cost, has emerged as a powerful tool for fine-scale forest resource monitoring [18,19]. When combined with deep learning algorithms, it exhibits remarkable potential for tree species identification [20]. Cheng systematically reviewed UAV-based semantic segmentation methods and demonstrated their widespread application in tree species classification and individual tree detection [21]. In single-modality studies, Liang utilized multi-angle UAV RGB imagery and an improved UNet++ to identify rubber tree trunks during the leafless period, achieving an F1-score of 94.7% [22]. Tang enhanced YOLOv5 with adaptive anchor boxes and global attention mechanisms, raising mPA to 94.5% and 88.2% at flight altitudes of 15 m and 30 m, respectively. In multispectral studies [23]. Li evaluated 8 semantic segmentation networks for tobacco seedling segmentation from UAV RGB images in Karst areas, with U-Net optimal in dams and U-Net++ best in hills [24]. Duan proposed the attention-enhanced CIA-UNet for single tree crown segmentation via UAV multispectral data, achieving 90.79% overall accuracy and 79.9% mIoU with multi-scale extraction and dual attention, outperforming mainstream models with efficient parameters [25]. Nevertheless, single-modality approaches still suffer from shadow interference and limited spectral discriminability in complex mountainous terrain.

Leveraging multi-source UAV data with cross-modal fusion has proven highly effective in overcoming these limitations. Xiao introduces a depth-attention gate that spatially aligns RGB textures with depth cues, enabling the network to maintain high saliency in low color-contrast areas and thus significantly reducing false positives [26]. Sothe concatenate hyperspectral “spectral fingerprints” with photogrammetric point-cloud and CHM-derived height metrics within an SVM framework; the joint spectral–structural feature space lifts the overall accuracy of 12 subtropical species by 15.4%, demonstrating that complementary physical and chemical signatures can break spectral confusion [27]. Baena bind red-edge spectra to UAV-SfM 3D objects through object-based analysis, allowing each classification unit to simultaneously capture leaf-level biochemical properties and crown-level geometric features, thereby raising species-mapping accuracy to 85.3% in a sparse, deciduous dry forest [28]. Lei fuse DSM multi-order gradients that capture fine crown surface undulations with spectral-texture-color self-similarity weights, iteratively merging over-segmented patches and delivering an F-score > 0.85 for individual crown delineation under dense, overlapping canopies [29]. Collectively, these studies demonstrate that multi-source UAV information fusion—through mechanisms such as attention weighting, joint feature spaces, object-level binding, or structure-guided spectral clustering—systematically enriches model discriminative capacity and dramatically enhances accuracy in complex environments.

In this study, we propose dual-branch cross-modal attention-enhanced UNet (DCA-UNet), a novel semantic segmentation framework that achieves deep integration of multi-source remote sensing data with superior accuracy and generalization capability. This research is justified by the limitations of existing methods, which often struggle with accurate crown detection in complex mountainous environments due to issues like shadow occlusion and complex backgrounds, necessitating advanced multimodal approaches to achieve UAV-based ginkgo crown monitoring in natural mountainous environments. Specifically, we design a dual-branch encoder (DBE) to independently extract modality-specific features from RGB and multispectral images. We then introduce a cross-modal interaction fusion module (CIF) that enables deep feature interaction and dynamic weighting between modalities. Finally, we develop an attention-enhanced decoder (AED) that aggregates multi-scale features using hybrid channel-spatial attention, significantly improving semantic consistency and boundary delineation. The proposed method provides a new technical pathway for monitoring wild ginkgo populations and offers a reliable, efficient solution for the conservation of endangered tree species and ecological restoration.

## 2. Materials and Methods

### 2.1. Study Region

The study area is located in the Tianmu Mountain National Nature Reserve, Hangzhou, Zhejiang Province, China (119°24′11′′~119°28′21′′ E, 30°18′30′′~30°24′55′′ N). Situated at the northern edge of the mid-subtropical zone and the core of the western Zhejiang mid-elevation hilly region, it is the only confirmed natural refuge for surviving wild Ginkgo in China. The reserve has an average elevation of 800–900 m (maximum 1506 m), annual mean temperature of 12–16 °C, annual precipitation of 1600–1800 mm, and relative humidity exceeding 80% year-round. Its deep, fertile acidic yellow soil provides a low-light, high-humidity, and well-drained microhabitat ideal for wild ginkgo, where trees exhibit height variations from approximately 15–30 m, with many over 300 years old, and the terrain features predominantly moderate to steep slopes ranging from 6° to 25°. The precise geographical coordinates and ground-surveyed data of Ginkgo trees within our study plots are archived and can be referenced against the extensive ecological records provided by GinkgoDB (https://ginkgo.zju.edu.cn/, accessed on 5 April 2025), an authoritative ecological genome database for Ginkgo [30].

### 2.2. Data Acquisition

**Figure 1:**
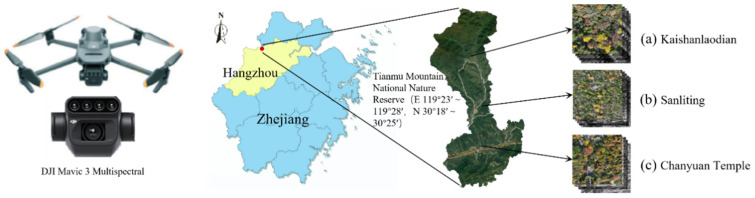
UAV Multispectral Data Acquisition in Tianmu Mountain National Nature Reserve.

Data were acquired using a DJI Mavic 3 Multispectral UAV platform (DJI, Shenzhen, China) equipped with a high-resolution visible-light (RGB) camera and a multispectral camera. Both the RGB camera and the individual multispectral sensors share a 4/3 CMOS form factor. The RGB camera is a 20-megapixel true-color sensor (5280 × 3956 pixels) with a 24 mm equivalent focal length, an aperture range of f/2.8 to f/11, and an ISO range of 100–6400. The multispectral camera comprises four co-registered 5-megapixel monochrome sensors (2592 × 1944 pixels), corresponding, from right to left, to Green (G: 560 ± 16 nm), Red (R: 650 ± 16 nm), RedEdge (RE: 730 ± 16 nm), and Near-Infrared (NIR: 860 ± 26 nm) bands (shown in Figure 1).

To specifically target wild ginkgo individuals during their leaf-coloring and leaf-fall stages, flight missions were conducted on three dates in November 2023: the 3rd, 11th, and 23rd. All flights took place between 11:00 and 15:00 local time under clear skies with sparse cloud cover and low wind speeds to ensure consistent illumination and minimize image distortion. To ensure the spatial independence of the evaluation data and mitigate potential data leakage, the flight mission was strategically designed. Data acquisition focused on three geographically independent core regions within Tianmu Mountain where wild ginkgo populations are concentrated: Kaishanlaodian (a), Sanliting (b), and Chanyuan Temple (c). The distances between these sites are approximately 2.5 km, 3 km, and 5 km, respectively. Additionally, imagery was specifically collected along a path between Sanliting and Kaishanlaodian to serve as an independent geographical source for the test set.

The UAV was operated in a ground-like flight mode following a pre-planned automated grid pattern. Flight parameters were set with a 70% forward overlap, 80% side overlap, a flight speed of 3 m/s, and a terrain-following altitude of 100 m. The image capture was triggered at equal distance intervals along the flight lines to ensure uniform coverage and high-quality data for subsequent orthomosaic generation.

### 2.3. Data Set Generation

**Figure 2:**
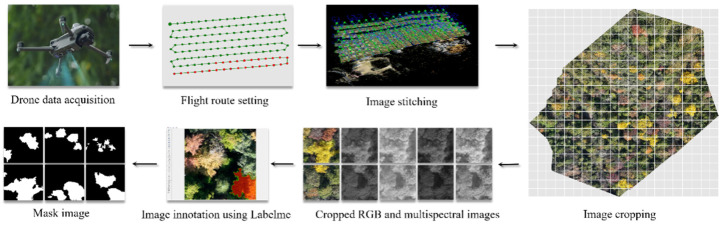
UAV Data Acquisition and Preprocessing Workflow.

The acquired raw UAV imagery was preprocessed and mosaicked using DJI Terra software (version 4.3.0, DJI, Shenzhen, China). The workflow included geometric correction, radiometric calibration, and image registration, ultimately generating high-resolution digital orthophoto maps (DOMs) for both RGB and the four multispectral bands (Green, Red, RedEdge, NIR). From these orthomosaics, image patches of 640 × 640 pixels were generated using a sliding window approach with a stride equal to 75% of the window size, resulting in a 25% overlap between adjacent patches. All cropped images were manually screened, and only those containing discernible ginkgo crowns were retained as valid samples (shown in Figure 2).

Pixel-level crown annotation was then performed to create the labels. To ensure the accuracy of annotation targets, the initial identification of ginkgo crowns within the orthomosaics was cross-verified using Quantum GIS (QGIS) software (version 3.38.0, QGIS.ORG, Laax, Switzerland) by overlaying the DOMs with available geographic coordinates of known ginkgo locations. Annotation was conducted on the RGB images using LabelMe software (version 3.16.7, labelme; GitHub; available at https://github.com/wkentaro/labelme, accessed on 15 April 2025), with ginkgo crown boundaries meticulously delineated. All annotation work was performed by graduate researchers within the team, and the resulting labels were subsequently reviewed and validated by an expert in forest remote sensing to guarantee consistency and correctness. To enhance model generalization and prevent overfitting, extensive data augmentation—including rotation, horizontal and vertical flipping, scaling, and cropping—was applied exclusively to the training set using OpenCV functions [31,32]. These operations substantially expanded the effective size and diversity of the training data, thereby improving model robustness.

Through the above process, a high-quality multimodal dataset for ginkgo tree crown recognition was constructed, comprising a total of 2961 valid image samples. Each sample integrates a high-resolution RGB image with its corresponding four multispectral band patches. The dataset was partitioned with explicit geographical separation, following the acquisition strategy in Section 2.2. Specifically, all 296 image samples from the independent flight path between Sanliting and Kaishan Laodian were designated as the test set for final evaluation. The remaining 2665 samples from the three geographically independent core regions (Kaishanlaodian, Sanliting, Chanyuan Temple) were randomly split into a training set and a validation set according to an 8:2 ratio, with the training set consisting of 2073 samples for model parameter optimization and feature learning, and a validation set of 592 samples for hyperparameter tuning and overfitting monitoring during the training process. This partitioning strategy ensures that model performance is assessed on geographically independent data.

### 2.4. Modeling Methods

#### 2.4.1. Dual-Branch Encoder (DBE)

Ginkgo crown segmentation in complex mountainous environments faces significant challenges, including high tree species diversity and severe canopy overlap. Single-modality data sources exhibit inherent limitations: although RGB imagery provides rich spatial texture, it lacks sufficient discriminative power for tree crowns with similar colors; multispectral imagery offers additional vegetation reflectance information but suffers from lower spatial resolution and limited capability to capture morphological details. Traditional multi-source fusion approaches often overlook feature heterogeneity between modalities, failing to fully exploit the complementary advantages of multimodal data and frequently resulting in information interference and loss of fine details.

To address these issues, we propose a dual-branch encoder (DBE). This module employs two independent ConvNeXt-Tiny backbones to separately process RGB and multispectral inputs, preserving modality-specific features while avoiding representation conflicts caused by early fusion [33]. The RGB branch receives three-channel visible-light images and focuses on extracting spatial texture features. The multispectral branch processes four bands (Red, NIR, Green, and RedEdge) to enhance the capture of vegetation physiological responses. To efficiently extend the channel dimension of the multispectral branch, we design a robust weight initialization strategy: the first three input channels of the initial convolution layer reuse ImageNet-pretrained weights, whereas the additional RedEdge channel is initialized using Gaussian distribution. This strategy enables effective knowledge transfer from visible to multispectral domains, maintains the representational capacity of the backbone, and circumvents slow convergence typically associated with training from scratch.

**Figure 3:**
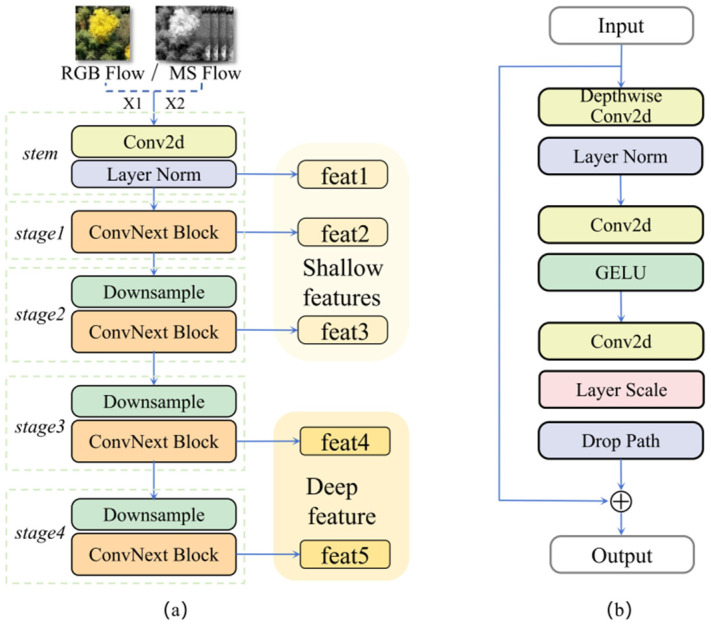
Architecture of the DBE module: (**a**) DBE Module with RGB or Multispectral Input; (**b**) ConvNeXt-Tiny Module.

Both branches progressively extract features using the ConvNeXt-Tiny hierarchical architecture [34,35]. The input images sequentially pass through the stem layer and multiple downsampling stages, where spatial resolution progressively decreases while the number of channels gradually increases. Training stability is maintained through GELU activation functions and LayerNorm, which normalizes along the channel dimension for each sample. Additionally, the architecture incorporates Layer Scale, which employs a learnable vector γ to scale features channel-wise and thereby enhances representational capability, as well as Drop Path, which randomly drops part of the residual branches to improve generalization ability. The RGB branch, with RGB Flow processing an RGB image as input, outputs five hierarchical feature maps (feat1–feat5), encompassing multi-scale information ranging from shallow textures to deep semantics. The multispectral branch, with MS Flow handling a multispectral image as input, extracts only deep feature maps (feat4 and feat5), focusing on high-level semantic features and vegetation physiological response differences while avoiding noise introduction from shallow features and reducing computational complexity (shown in Figure 3). This design ensures the RGB branch specializes in spatial detail extraction while the multispectral branch highlights spectral response differences, establishing a foundation for subsequent cross-modal interaction.

#### 2.4.2. Cross-Modal Interactive Fusion (CIF)

In multimodal remote sensing image segmentation tasks, conventional feature fusion strategies lack explicit cross-modal interaction, failing to effectively integrate the spatial details of RGB imagery with the spectral reflectance characteristics of multispectral data. This often results in edge blurring and spectral confusion in ginkgo crown recognition. To address this limitation, we propose a Cross-Modal Interactive Fusion (CIF), which significantly enhances recognition accuracy through efficient cross-modal interaction and adaptive fusion.

**Figure 4:**
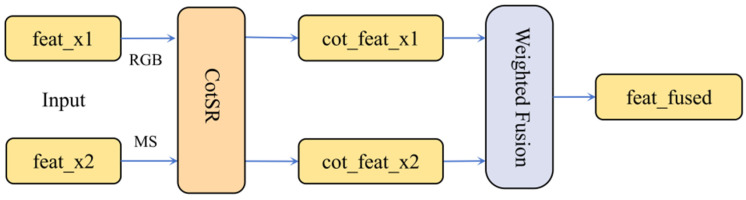
Architecture of the CIF module.

The CIF employs a cascaded structure where Cross-modal Spatial Reasoning (CotSR) modules are deployed at high-level feature layers (feat4 and feat5), followed by feature integration through a dynamic weight fusion module to generate fused feature maps (shown in Figure 4). In this structure, ‘feat’ refers to the inputs, specifically feat4 or feat5 from the respective branches; ‘cot_feat’ represents the output after cross-modal interaction via CotSR; and ‘feat_fused’ denotes the output after fusion via the WeightFusion module. The CotSR module, designed based on the dual-input cross-attention mechanism from DANet [36], is implemented at feat4 and feat5 feature layers to enable interactive enhancement between deep features from RGB and multispectral branches. Deep features possess larger receptive fields and stronger semantic abstraction capabilities, effectively avoiding interference from detail noise present in shallow features, while their lower spatial resolution significantly reduces computational overhead for attention matrices. By explicitly capturing spatial dependencies between modalities, the CotSR utilizes RGB texture details to optimize multispectral responses; simultaneously, the spectral features provided by the multispectral branch effectively enhance the RGB branch’s capability to distinguish ginkgo from background vegetation, thereby significantly improving complementary information transmission efficiency through bidirectional pathways.

CotSR takes feature maps from two branches as input. Each branch first generates its own Query (Q), Key (K), and Value (V) representations through 1 × 1 convolutions: R_Q_, R_K_, R_V_ for the RGB branch, and M_Q_, M_K_, M_V_ for the Multispectral branch.

**Figure 5:**
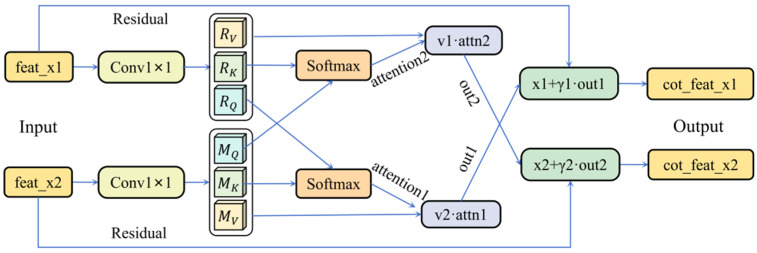
Architecture of the CotSR module.

Cross-attention is applied between the branches to enable inter-modal interaction. For the RGB branch, R_Q_ interacts with the M_K_ to compute a similarity matrix. This matrix is normalized by Softmax and then used to weight the M_V_, producing a complementary feature component. The same process is applied symmetrically for the Multispectral branch, where M_Q_ interacts with the R_K_ and R_V_. Finally, the original features from each branch are fused with their corresponding complementary components via residual connections, yielding interactively enhanced features cot_feat_x1 and cot_feat_x2 (shown in Figure 5). This process is defined by Formulas (1) and (2).

feat_out_1_ = X_1_ + γ_1_∙ (V_2_∙ (Softmax (Q_1_∙K_2_))^T^) (1)

feat_out_2_ = X_2_ + γ_2_∙ (V_1_∙ (Softmax (Q_2_∙K_1_))^T^) (2)

where X denotes the original branch features, γ represents a learnable scalar parameter. The Softmax operation normalizes the attention weight matrix. This mechanism facilitates inter-modal information exchange through cross-attention, ultimately integrating original features with enhanced components via residual connections, thereby ensuring progressive learning and preventing overfitting.

Building upon the features enhanced by CotSR, this study further introduces a dynamic weight fusion module to adaptively weight and fuse RGB and multispectral features [37,38], outputting a single fused feature map Feat_fused (shown in Figure 4). The computation is defined as follows:

F_fused_ = α∙F_RGB_ + β∙F_MS_ (3)

where α and β are learnable parameters. During forward propagation, the weights are adaptively adjusted through these learnable parameters α and β, dynamically balancing the contributions from different modalities. This approach avoids dimensional expansion, maintains computational efficiency, and thereby enhances fusion flexibility and recognition accuracy.

This module performs fusion on the CotSR-enhanced feature maps, taking cot_feat_x1 (RGB) and cot_feat_x2 (multispectral) as inputs and outputting the fused feature map feat_fused. Its processing pipeline is concise and efficient, utilizing learnable parameters to dynamically adjust the fusion ratio between RGB and multispectral features. This enables the model to adaptively optimize feature representation based on the characteristics of the input data. The output fused feature map not only preserves spatial details from RGB images but also enhances vegetation physiological response information from multispectral data, providing a more discriminative multimodal feature foundation for subsequent decoder segmentation. Through explicit cross-modal interaction and dynamic weight allocation, this design effectively addresses issues of information interference and computational redundancy in traditional fusion methods, significantly improving the robustness of ginkgo tree crown identification in complex mountainous environments.

#### 2.4.3. Attention-Enhanced Decoder (AED)

Traditional decoders typically rely solely on transposed convolution or bilinear interpolation to restore the spatial resolution of feature maps, lacking an effective mechanism for filtering the fused features. This approach tends to introduce noise and lose critical semantic information during the upsampling process, consequently leading to blurred segmentation boundaries and reduced model robustness. To address these limitations, this paper designs AED. Building upon the classical U-Net architecture, the AED deeply integrates the convolutional block attention module (CBAM) to progressively reconstruct high-resolution, semantically strong segmentation prediction maps from the advanced fused features output by the CIF module. Through the synergistic combination of layer-wise upsampling, skip connections, and the CBAM attention mechanism, the AED achieves efficient integration of multi-scale features [39], effectively avoids semantic dilution, and significantly enhances both the segmentation accuracy of tree crown boundaries and semantic consistency.

**Figure 6:**
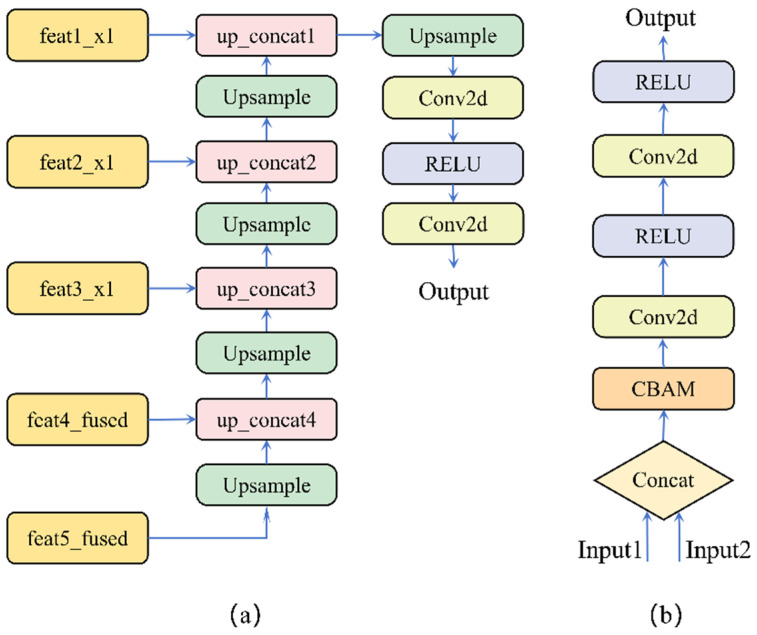
Architecture of the AED: (**a**) Overall structure of AED; (**b**) Structure of the up_concat module within AED.

The AED achieves multi-scale feature integration through layer-wise upsampling and skip connections. The decoding process begins with the high-level semantic features (feat5_fused) output from the CIF module. These features first undergo a 2× upsampling operation and are subsequently concatenated with the fused features (feat4_fused) at the corresponding scale. The decoder then progressively incorporates the corresponding shallow or intermediate features (feat3_x1, feat2_x1, feat1_x1) from the RGB branch at each subsequent stage (shown in Figure 6a). This design strategy effectively leverages the high-resolution spatial details preserved by the RGB branch to optimize boundary reconstruction, while deliberately avoiding potential spectral noise and computational redundancy that may exist in the shallow features of the multispectral branch.

The Up-Concat module serves as the core component of the AED for refined feature fusion(shown in Figure 6b). This module accepts two inputs: the upsampled features from the previous decoding stage, and the encoder features at the corresponding scale introduced via skip connections. These two inputs are initially concatenated along the channel dimension to integrate contextual information. Rather than being directly fed into convolutional layers, the concatenated features first undergo adaptive refinement through a CBAM attention module.

**Figure 7:**
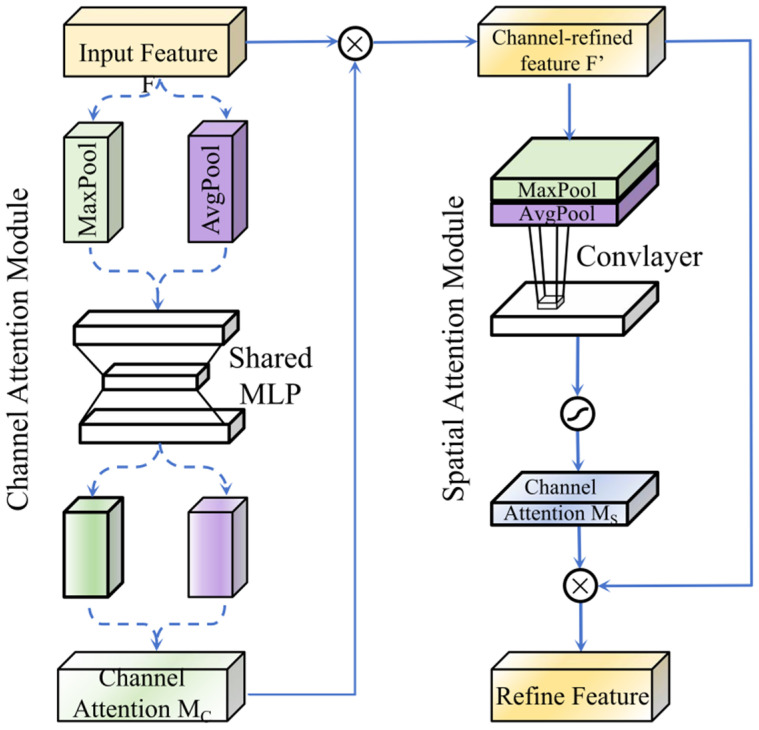
Detailed structure of the CBAM.

The CBAM automatically evaluates and weights the importance of each channel and spatial position in the feature map through its parallel channel and spatial attention sub-module (shown in Figure 7) [40]. The attention-filtered features are then processed by a convolutional block for non-linear transformation and dimensionality reduction, ultimately producing the refined feature map for that particular decoding stage.

The channel attention mechanism aims to model the importance of different feature channels. Its core operation leverages both global average pooling and global max pooling to aggregate spatial information and generates channel-wise weights through a shared Multi-Layer Perceptron (MLP) (see Equation (4)). Mc represents the final channel attention map, X denotes the input feature, and σ represents the Sigmoid function.

M_c_ = σ (MLP (AvgPool (X) + MLP (MaxPool (X))) (4)

The spatial attention module further focuses on informative spatial regions. It applies both average pooling and max pooling operations along the channel axis, concatenates the resulting two 2D spatial descriptors, and generates the spatial attention weights by processing the concatenated result through a 7 × 7 convolutional layer (see Equation (5)). Ms represents the final spatial attention map, f 7 × 7 denotes a convolutional layer with a 7 × 7 kernel, and [;] represents the channel-wise concatenation operation.

M_s_ = σ (f^7×7^([AvgPool(X); MaxPool(X)])) (5)

The overall application of CBAM is expressed in Equation (6). The output feature (X′) is obtained by the element-wise multiplication (⊙) of the X, Mc, and Ms, progressively refining the feature representation across both channel and spatial dimensions.

X′ = X ⊙ M_c_ ⊙ M_s_ (6)

The three-stage “concatenation-attention recalibration-convolutional transformation” pipeline effectively filters critical features while suppressing irrelevant responses. It ensures continuous enhancement of information quality throughout the resolution recovery process, thereby significantly boosting feature representation capability and segmentation accuracy in complex scenarios.

### 2.5. Model Workflow

**Figure 8:**
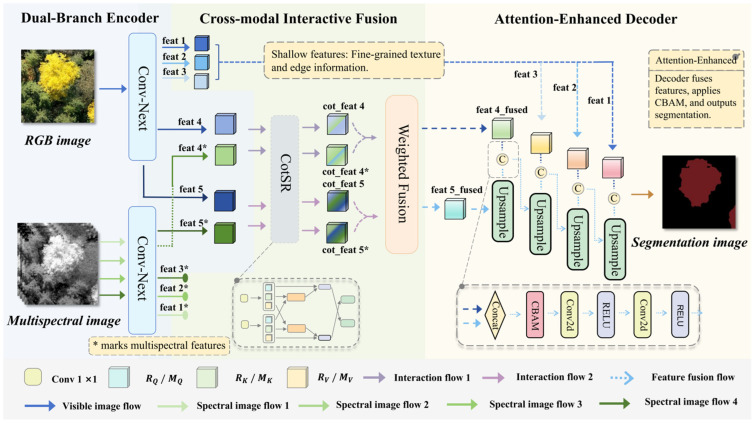
Overall architecture of the proposed DCA-UNet.

To effectively integrate RGB and multispectral features while enhancing hierarchical semantic perception, this paper proposes a novel DCA-UNet model. The input images are processed in parallel through the DBE handling heterogeneous inputs: the RGB branch outputs five hierarchical feature maps (feat1–feat5), while the multispectral branch generates two deep-level feature maps (feat4, feat5), preserving multi-scale semantic information. The deep features are collaboratively optimized by the CIF module, which employs dual-branch cross-attention mechanisms through its CotSR units at key stages. This process calculates inter-modal correlation weights via Query-Key computation and performs weighted fusion using learnable parameters α and β to produce compact unified features, thereby enhancing multi-source information integration efficiency. The decoding phase adopts the AED that progressively restores resolution through four upsampling stages. Each stage incorporates feature concatenation followed by CBAM processing, which sequentially recalibrates channel weights and spatially focuses on critical regions, with dual convolutional layers refining the features before ultimately generating the output segmentation map (shown in Figure 8).

### 2.6. Evaluation Indicators

To comprehensively and objectively evaluate the performance of DCA-UNet, four widely adopted metrics were employed: IoU (Intersection over Union), PA (Pixel Accuracy), Precision, and F1-score [41,42,43,44]. IoU measures the overlap between predicted and labels, calculates the ratio of True Positives (TPs) and the total number of positive predictions, which includes TP, False Positives (FPs) and False Negatives (FNs), directly reflecting boundary delineation accuracy (see Equation (7)). PA represents the proportion of correctly classified pixels, which includes TP and True Negatives (TN), indicating the overall discriminative capability of the mode (see Equation (8)). Precision quantifies the fraction of true ginkgo crown pixels among all, calculates the ratio of TP and the total number of positive predictions, which includes TP and FP (see Equation (9)). F1-score, the harmonic mean of Precision and Recall, provides a balanced comprehensive evaluation in the presence of class imbalance, ensuring an optimal trade-off between missed detections and false alarms (see Equation (10)).

(7) $$\mathrm{IoU}=\frac{TP}{TP+FP+FN}\times 100\%$$

(8) $$\mathrm{PA}=\frac{TP+TN}{N}\times 100\%$$

(9) $$\mathrm{Precision}=\frac{TP}{TP+FP}\times 100\%$$

(10) $$F1\text{-}\mathrm{score}=\frac{2\times Precision\times Recall}{Precision+Recall}\times 100\%$$

### 2.7. Experiment Settings

The experiments were conducted on a workstation equipped with dual NVIDIA GeForce RTX 3060 Ti GPU (8 GB VRAM per card). The hardware environment consisted of an Intel^®^ Core™ i7-12700K processor with 128GB RAM, while the software environment was based on a Windows 10 64-bit operating system. The algorithm implementation utilized Python 3.8 (Anaconda distribution) and PyTorch 1.13.1 framework, with CUDA 11.6 enabled for GPU acceleration.

This study employed ConvNeXt-Tiny as the backbone network. The model weight initialization strategy was configured as follows: the first three channels (Red, NIR, Green) for both RGB and multimodal inputs directly inherited pre-trained weights from ImageNet, while the fourth multispectral channel (RedEdge) was randomly initialized using a Gaussian distribution (standard deviation 0.01), thereby achieving knowledge transfer from visible to multispectral domains. During the training phase, the Adam optimizer was adopted (β_1_ = 0.9, β_2_ = 0.999, weight decay 1 × 10^−4^) with an initial learning rate of 1 × 10^−4^. Automatic Mixed Precision (AMP) training was enabled, and a cosine annealing strategy was implemented to reduce the learning rate from 1 × 10^−4^ to 1 × 10^−6^ over 200 epochs, with early stopping triggered if the validation mIoU showed no improvement for five consecutive epochs. The batch size was set to 4, using cross-entropy as the loss function.

### 2.8. Baseline Models and Comparison Setup

To validate the effectiveness of the proposed DCA-UNet framework, we first established baseline models for comparison in the context of ginkgo tree crown segmentation from UAV remote sensing imagery in natural mountainous forests. This task involves complex factors such as background vegetation interference, uneven lighting, shadow occlusion, and crown overlapping.

We selected UNet as the primary baseline due to its U-shaped encoder–decoder architecture and multi-scale skip connections, which enable the fusion of low-level spatial details and high-level semantic features. This makes it suitable for handling noisy conditions in both RGB and multispectral images. To provide a comprehensive evaluation, additional classical semantic segmentation models were chosen as baselines: PSPNet, DeepLabv3, and SegNet. These models were selected because they represent foundational approaches in semantic segmentation with widespread use in remote sensing and vegetation analysis tasks [45,46,47,48,49,50,51,52].The reasons for their inclusion are their complementary strengths: PSPNet excels in multi-scale context aggregation, DeepLabv3 in atrous convolutions for dense feature extraction, and SegNet in efficient encoder–decoder designs for real-time applications. For single-modality baselines, models were trained on RGB inputs and multispectral data separately to highlight limitations. For multimodal comparisons, we adapted them by deep feature fusion of RGB and multispectral data, and evaluated on the ginkgo dataset with both inputs to assess fusion benefits.

The core advantage of UNet over other models lies in its unique U-shaped encoder–decoder architecture and multi-scale skip connections, which enable the fusion of low-level spatial details and high-level semantic features, making it suitable for robust segmentation in complex scenarios. To further optimize the backbone within UNet, we compared diverse designs from parameter-efficient to high-capacity architectures, including ResNet18, ResNet34, ResNet50, VGG16, MobileNetv3, and ConvNeXt-Tiny [53,54,55,56,57]. These were selected to cover a range of complexities, with ConvNeXt-Tiny chosen as the primary backbone due to its balance of efficiency and performance, stemming from its design that effectively integrates advantages from both convolutional and Transformer architectures.

To address the challenges of shadow occlusion and complex backgrounds in ginkgo tree crown recognition, we introduced the CIF module for efficient cross-modal interaction and adaptive fusion. The CotSR attention mechanism employed in this study effectively captures spatial dependencies between modalities. This was followed by a dynamic weight fusion module using learnable parameters α and β to adaptively balance RGB and multispectral contributions, avoiding dimensional expansion and computational redundancy while providing more robust multimodal feature representations for subsequent segmentation decoders.

Based on the DBE and CIF modules, the Attention-Enhanced Decoder (AED) was designed to progressively reconstruct high-resolution segmentation maps. It integrates layer-wise upsampling, skip connections, and attention mechanisms to optimize feature fusion, thereby providing a refined structural foundation for the performance validation in the following results section.

## 3. Results and Analysis

### 3.1. Baseline Model and Backbone Network Comparison

The test results (shown in Table 1) indicate that under RGB input conditions, UNet achieved an IoU of 91.56%, PA of 96.28%, Precision of 95.77%, and F1-score of 95.99%, outperforming all other models. When the input is switched to multispectral imagery, all models exhibit performance degradation, with IoU dropping by 4–15% across the board. However, UNet achieves an IoU of 87.59%, experiencing only a 4.0% decline, thus demonstrating superior robustness.

**Figure 9:**
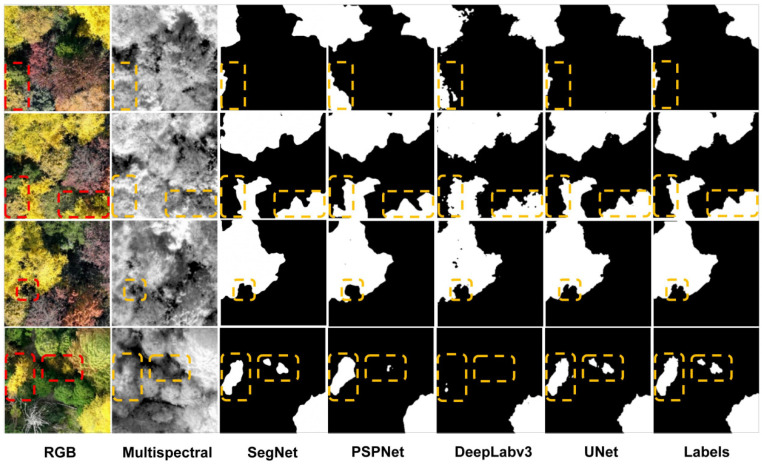
Visualization of segmentation masks obtained by different models on the ginkgo dataset.

**Figure 10:**
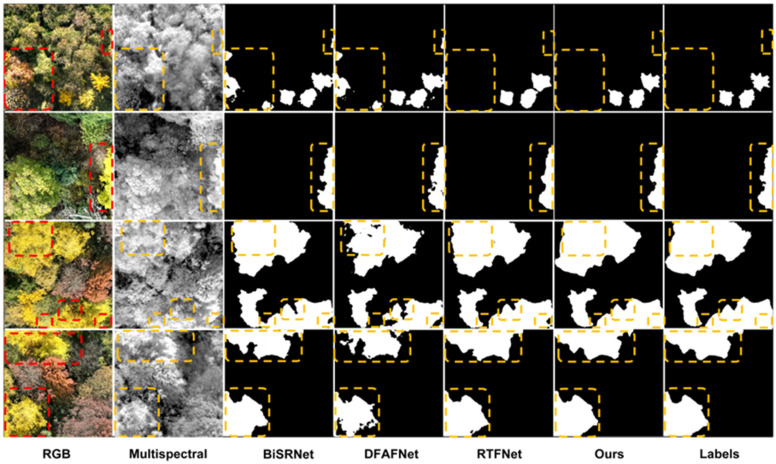
Comparison of segmentation masks obtained with different multimodal models.

**Figure 11:**
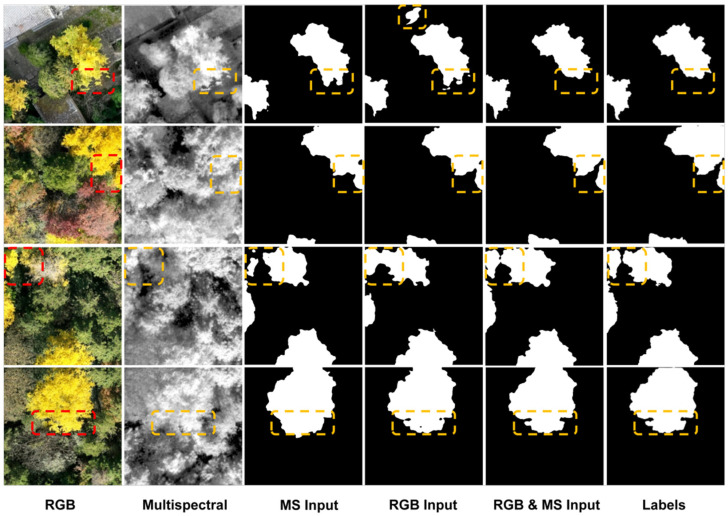
Comparison of segmentation masks obtained with different input modalities.

**Figure 12:**
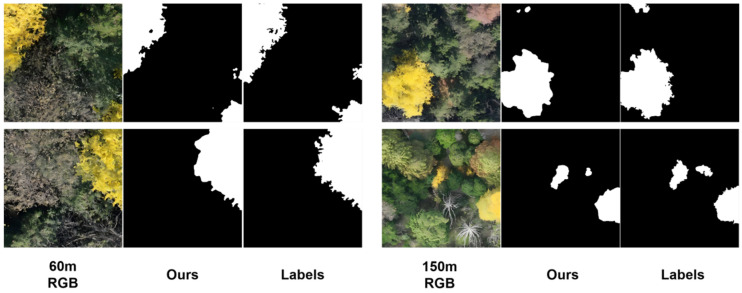
Visualization of segmentation results for UAV images at different flight heights.

Visualizations of segmentation results across different scenarios reveal performance differences among models (shown in Figure 9). In the first row, both SegNet and PSPNet misidentified non-ginkgo canopy in the lower left corner as ginkgo crowns. The second row shows that PSPNet and DeepLabv3 incorrectly incorporated adjacent tree crowns into the ginkgo segmentation area. Although the third row contained minimal interference, PSPNet still failed to completely segment the boxed ginkgo canopy in the lower portion. In the fourth row, small-canopy ginkgo trees in shaded areas were missed by both PSPNet and DeepLabv3. These results demonstrate that under challenging conditions including color interference, structural variations, and shadow occlusion, UNet more accurately distinguishes ginkgo canopies from background elements, significantly reducing both mis-segmentation and missed detection. For clarity in the visualization figures (Figure 9, Figure 10, Figure 11 and Figure 12), the white areas represent the model-predicted segmentation masks for ginkgo canopies, while the black regions denote the background. The “Labels” column displays the manually annotated reference masks from the validation dataset. Yellow and red dashed boxes are used to highlight regions where the models exhibit mis-segmentation or missed detection, serving as visual cues for performance comparison.

Experimental results show that ConvNeXt-Tiny outperformed other backbone networks across all metrics, achieving IoU 92.26%, PA 95.59%, Precision 95.88%, and F1-score 95.96% (shown in Table 2). These findings validate its enhanced global context modeling and anti-interference capabilities in complex forest scenarios.

To overcome the limitations of single data sources, we introduced multispectral data and constructed a DBE. After integrating the dual-branch input structure with the baseline model, compared to using only single RGB data source, the IoU, PA, Precision, and F1-score increased to 92.32%, 95.69%, 95.97%, and 96.09%, respectively. This confirms the potential of multimodal fusion.

### 3.2. Evaluation of Cross-Modal Interaction Fusion (CIF) Module

To validate the effectiveness of the proposed CIF module (integrating CotSR and dynamic weight fusion), we compared it with two state-of-the-art multimodal fusion frameworks, namely DDFNet and MCANet [38,58,59,60,61]. DDFNet emphasizes bidirectional feature flow, whereas MCANet focuses on multi-channel attention mechanisms to enhance local detail modeling. The experimental results demonstrate that CIF achieved an IoU of 93.28%, outperforming DDFNet (92.89%) and MCANet (92.37%). It also attained the highest scores across other metrics, including PA, Precision, and F1-score (shown in Table 3). These results indicate that CIF can more effectively integrate complementary information from dual modalities, mitigating issues of spectral confusion and boundary ambiguity, with the dynamic weighting providing additional refinements in complex scenarios.

To further demonstrate the adaptive nature of the dynamic weight fusion within CIF, we analyzed the learned weights (α for RGB and β for multispectral) across different fusion stages (shown in Table 4).

In feat4, the RGB weight (α = 1.107) is higher than the multispectral weight (β = 0.971), suggesting greater emphasis on spatial details from RGB for boundary delineation. In contrast, feat5 shows a slight preference for multispectral features (β = 0.915 > α = 0.892), highlighting the model’s adaptation to leverage spectral information for semantic discrimination in complex environments.

The CIF module enables deep inter-modal interaction via CotSR, capturing spatial-spectral dependencies. The dynamic weight fusion adaptively integrates dual-modal features, addressing traditional methods’ deficiencies in interaction and adaptability while maintaining efficiency.

### 3.3. Evaluation of Attention-Enhanced Decoder (AED) Module

Building upon the classical UNet decoding architecture, the AED incorporates the CBAM attention mechanism to enable feature selection during the process of gradually upsampling high-level fused features from the CIF module and integrating them with shallow and intermediate RGB features. Through sequential upsampling and skip connections, the AED aggregates multi-scale features, and employs the CBAM for adaptive refinement after each feature concatenation. This sequential channel and spatial attention mechanism effectively filters critical features while suppressing irrelevant noise, thereby optimizing boundary precision and enhancing semantic consistency.

To validate the necessity of the CBAM in the AED [40], we comparing model performance with and without CBAM incorporation (shown in Table 5). Experimental results reveal that incorporating the CBAM attention mechanism consistently improves all evaluation metrics. Although the absolute gains are modest, such enhancement remains highly valuable given that the baseline performance already approaches the theoretical ceiling.

### 3.4. Comparative Evaluation and Ablation Analysis of DCA-UNet

To validate the effectiveness and advancement of DCA-UNet, we compare it with three models, binarized spectral-redistribution network (BiSRNet), RGB-thermal fusion network (RTFNet), and differential feature attention fusion network (DFAFNet). These models can reflect the performance gains brought by integrated attention mechanisms and cross-modal fusion. BiSRNet, a binarized multimodal model for semantic segmentation, serves as an efficiency benchmark in multimodal remote sensing, particularly for high-resolution image processing [62]. RTFNet provides a classic reference as an authoritative method for RGB-auxiliary modality fusion and feature integration in remote sensing semantic segmentation [63]. DFAFNet, a recent multimodal model emphasizing differential feature attention fusion for multi-source remote sensing images [64], evaluates DCA-UNet’s competitiveness against state-of-the-art approaches in vegetation and crown recognition. Experimental results demonstrate that DCA-UNet achieves significant performance improvements in ginkgo crown recognition.

With fused RGB-multispectral input, DCA-UNet achieved optimal results across all metrics, attaining IoU, PA, Precision, and F1-score of 93.42%, 96.82%, 96.38%, and 96.60%, respectively (shown in Table 6). In comparison, BiSRNet yielded 88.98%, 93.37%, 92.75%, and 92.50%; RTFNet achieved 91.13%, 95.88%, 95.09%, and 95.39%; while DFAFNet obtained 81.23%, 92.45%, 91.76%, and 89.65%. These results clearly indicate that DCA-UNet, by integrating DBE, CIF, and AED, significantly enhances multi-source feature extraction and fusion, thereby outperforming baselines in accuracy and robustness, particularly in complex multimodal scenarios.

Following the same visual convention established earlier, we compared the segmentation performance of three multimodal models across four representative scenarios to assess the model’s recognition accuracy, particularly for small targets and shadowed areas: small targets, edge targets, complex environments, and shadow occlusion (shown in Figure 10). In these cases, BiSRNet exhibited misidentification in regions with similar colors; DFAFNet performed poorly under similar colors and shadow occlusion; RTFNet handled color similarities adequately but struggled with shadows. In contrast, our DCA-UNet achieved precise segmentation across all scenarios, effectively capturing contour details, handling shadow occlusion, distinguishing similar colors, and managing complex environments. This demonstrates DCA-UNet’s superior robustness and accuracy in challenging multimodal ginkgo crown recognition tasks compared to the baselines.

To validate the effectiveness of multimodal input and cross-modal fusion in enhancing model performance, this section compares DCA-UNet’s performance using only RGB, only multispectral, and combined fusion (RGB+MS) as input. With the model architecture fixed, different input modalities yielded varying results: RGB input achieved an IoU of 87.67%, PA of 95.41%, Precision of 91.53%, and F1-score of 93.43%; multispectral input yielded 87.41%, 91.03%, 85.14%, and 87.99%, respectively; while multimodal fused input improved all metrics to optimal levels, achieving 93.42%, 96.82%, 96.38%, and 96.60%, respectively (shown in Table 7). This comparison demonstrates that, within the structurally optimized DCA-UNet framework, introducing and fusing multispectral information leverages the complementary advantages of different modal data, thereby significantly enhancing the model’s segmentation capability.

Using the visualization scheme described above, this study selected four representative scenarios to comparatively analyze the segmentation performance of RGB input, multispectral input, and their fused input in ginkgo tree crown identification tasks (shown in Figure 11), thereby validating the effectiveness of the multimodal fusion strategy. As shown in the figure, RGB input exhibited misidentification of non-canopy structures (e.g., rooftops) but performed well in delineating canopy gaps and contours. Multispectral input demonstrated advantages in distinguishing surface features but struggled with edge details of small targets. In contrast, the fused input consistently captured crown contours and structural details more accurately across all scenarios, achieving optimal segmentation performance. These results indicate that cross-modal fusion effectively combines the complementary strengths of RGB and multispectral data, significantly enhancing segmentation accuracy and robustness under varying complexities. This confirms the necessity and importance of the proposed multimodal input strategy for ginkgo crown recognition.

### 3.5. Generalization Ability Evaluation

In practical drone monitoring scenarios, the training conditions of models may vary. Factors such as flight altitude and image resolution often differ from the training data, which can affect the stability of model performance. Therefore, a systematic evaluation of the model’s generalization capability is crucial.

The core dataset of this study was collected by a drone conducting terrain-following flights at a baseline altitude of 100 m. To thoroughly investigate the generalization performance of the DCA-UNet model against variations in flight altitude, additional data were acquired at 60 m and 150 m. A specialized testing protocol was designed: the model was first trained on the 100 m data until performance stabilized (reaching an IoU of 93.42%). Its parameters were then fixed, and it was directly evaluated for generalization on three independent test sets: 60 m, 150 m, and a mixed set combining both [65].

On the baseline altitude (100 m) test set, the model achieved excellent segmentation performance, with IoU, PA, Precision, and F1-score reaching 93.42%, 96.82%, 96.38%, and 96.60%, respectively. In the 60 m low-altitude scenario, the respective metrics were 90.59%, 94.06%, 96.09%, and 95.06%, indicating the model’s good adaptability to the scale and perspective changes induced by the lower altitude. Facing the inherent challenges of reduced target size and blurred details in the 150 m high-altitude scenario, the model maintained strong robustness, achieving an IoU of 89.22% and an F1-score exceeding 94%, effectively validating its capability to handle domain shift problems caused by altitude variations. As shown in Figure 12, which presents segmentation masks for representative UAV images at 60 m and 150 m altitudes, although the model’s performance exhibits a slight decline compared to the baseline due to variations in resolution and detail, it nonetheless successfully captures the fundamental contours and structures of ginkgo crowns, ensuring reliable basic recognition even under suboptimal conditions. Furthermore, to simulate practical application environments with dynamically changing altitudes, a mixed test on the combined 60 m and 150 m data showed that the model achieved a comprehensive performance of IoU 89.43% and F1-score 94.42% (shown in Table 8). This result surpasses the performance on the 150 m data alone and approaches the level achieved on the 60 m data, demonstrating that the model can effectively integrate complementary features from different altitudes, thereby maintaining stable and reliable detection performance in complex and variable real-world scenarios.

## 4. Discussion

This study proposes and validates DCA-UNet, an innovative architecture utilizing UAV remote sensing imagery for multimodal ginkgo crown segmentation in complex mountainous forest environments. Experimental results demonstrate that DCA-UNet significantly outperforms single-modal models such as UNet, BiSRNet, and DeepLabv3 in recognition accuracy with fused RGB-multispectral inputs, while also exhibiting substantial advantages over multi-modal models like BiSRNet, RTFNet, and DFAFNet. The IoU, PA, precision, and F1-score reached 93.42%, 96.82%, 96.38%, and 96.60%, respectively. particularly excelling in handling spectral variations and ginkgo crown boundary precision during autumn phenological stages.

Compared to representative existing methods, the proposed DCA-UNet exhibits distinctive value in architectural design and modality fusion. First, Mask R-CNN-based methods are confined to single optical modalities, performing poorly with non-optical data like LiDAR, which reflects the inherent limitation of their input channel modification in enabling deep spectral-structural cross-modal interactions [66]. Second, hybrid LiDAR-focused segmentation approaches only achieve superficial cross-modal integration, failing to fully exploit the complementarity between optical and LiDAR data in complex mountainous areas [67,68]. Third, BiSRNet supports multi-source data but lacks deep cross-modal interactions, thus underutilizing the complementary information between RGB and multispectral data in such scenarios and constraining segmentation accuracy. Additionally, the recently proposed two-stage segment-then-classify (STC) strategy performs well in specific phenological periods but relies on single RGB modality, imposing fundamental bottlenecks in distinguishing color-similar species or overcoming shadow occlusions due to limited data source diversity [69]. In summary, these models exhibit deficiencies in modality adaptability, fusion depth, and data source variety.

The DCA-UNet developed in this study achieves systematic optimization through its synergistic architectural design. The model first employs the DBE to independently encode RGB and multispectral data, effectively preserving and enhancing modality-specific information to establish a pure and rich feature foundation for subsequent fusion. The CIF module, centered on the CotSR component, enables deep bidirectional feature interactions and adaptively balances modality contributions via a dynamic weighting mechanism, overcoming defects in models like BiSRNet, such as shallow interactions and fixed fusion strategies, and significantly alleviating spectral confusion and edge ambiguity. In the decoding phase, the AED incorporates the CBAM attention mechanism, which autonomously selects key semantic information and suppresses background noise during multi-scale feature upsampling and fusion, thereby precisely restoring crown contours in complex canopy backgrounds and improving segmentation boundary precision and semantic consistency. In summary, DCA-UNet systematically addresses key challenges in multimodal adaptation, deep feature fusion, and detail reconstruction through the collaborative operation of DBE, CIF, and AED modules, enabling robust and high-precision ginkgo crown segmentation.

The study successfully verifies the high-precision segmentation capability of the proposed DCA-UNet framework for autumn ginkgo phenological monitoring, demonstrating the synergistic advantage of integrating multimodal data with a dedicated network architecture. Its superior performance is primarily validated on data from the coloration and leaf-fall stages, where distinctive spectral and color features are most pronounced. For optimal accuracy, image acquisition between 11:00 and 14:00 local time is recommended to ensure sufficient illumination and higher solar angles, thereby minimizing shadow occlusion. A key strength of the method is its robustness under suboptimal conditions; by fusing RGB and multispectral information, it significantly improves recognition accuracy for small targets and within shadowed regions. This effectively reduces interference from adjacent tree crowns, enhancing detection reliability in complex field environments. For practitioners, this method offers an efficient, automated alternative to labor-intensive manual surveys, enabling scalable and repeatable crown segmentation to support species management and ecological studies.

Nevertheless, a limitation lies in the dynamic fusion module’s use of global learnable scalars (α and β) at each stage. While computationally efficient and interpretable, this design does not fully account for spatial or channel-wise variations in feature importance, potentially limiting adaptive fusion in heterogeneous scenes. To extend the model’s applicability and robustness, future work will focus on: (1) developing spatially and channel-wise adaptive fusion mechanisms for more dynamic, context-aware multimodal integration; (2) constructing cross-phenological datasets and employing generalization techniques like domain adaptation to address high feature similarity across growth stages; and (3) integrating additional data sources such as hyperspectral and LiDAR to enhance the model’s universality and comprehensive monitoring efficacy.

## 5. Conclusions

This study proposes DCA-UNet, a novel semantic segmentation framework specifically designed for precise crown delineation of wild ginkgo in mountainous environments. To address the scarcity of remote sensing data for wild ginkgo, we constructed a high-quality multimodal dataset comprising co-registered RGB and multispectral images acquired from a UAV platform. The proposed DCA-UNet employs the DBE that processes RGB and multispectral inputs in parallel through independent downsampling paths, effectively extracting and preserving multi-scale modality-specific features to establish a robust foundation for subsequent deep fusion. We further designed a novel CIF that promotes deep feature interaction across modalities and applies learnable dynamic weighting, significantly enhancing the integration of complementary multi-source information. At the decoding stage, the AED progressively restores high-resolution details via layered upsampling while leveraging hybrid channel-spatial attention to selectively emphasize informative features, thereby ensuring superior boundary accuracy and semantic consistency. Experimental evaluation demonstrates that DCA-UNet achieves outstanding performance across all key metrics, with the PA of 96.82%, Precision of 96.38%, IoU of 93.42%, and F1-score of 96.60%. Compared with the single-modality RGB baseline, DCA-UNet improves IoU and Precision by 5.75% and 4.85%, respectively. The performance gain is even more pronounced against the multispectral-only baseline, with improvements of 6.01 and 11.24% in IoU and Precision. Compared to cross-modal, these results outperform DFAFNet by 12.19%, 6.37%, 4.62%, and 6.95%, respectively. These results unequivocally validate the superior multimodal fusion capability of DCA-UNet and its effectiveness and advancement in accurate ginkgo crown segmentation. The proposed framework provides a practical and efficient technical solution for high-precision monitoring of endangered forest tree species, offering significant potential for biodiversity conservation and ecological restoration.

## Figures and Tables

**Table 1:** Performance evaluation of different models on the ginkgo dataset.

Input	Model	Parameter (%)			
		IoU	PA	Precision	F1
RGB	SegNet	85.45	91.30	91.17	90.79
	PSPNet	87.33	93.79	92.60	92.88
	DeepLabv3	90.65	95.91	94.29	95.29
	UNet	91.56	96.28	95.77	95.99
MS	SegNet	76.04	82.30	83.71	83.84
	PSPNet	76.22	82.74	90.63	86.50
	DeepLabv3	78.03	83.05	92.80	87.65
	UNet	87.59	93.61	93.85	93.53

**Table 2:** Performance comparison of different backbone networks with UNet.

Model	Parameter (%)			
	IoU	PA	Precision	F1
ResNet18	91.87	95.03	95.40	94.76
ResNet34	83.90	87.09	94.81	91.25
ResNet50	92.16	95.27	95.76	95.78
VGG16	91.56	96.28	95.77	95.99
Mobilenetv3	90.36	94.05	95.76	94.85
ConvNeXt-Tiny	92.26	95.59	95.88	95.96

**Table 3:** Performance comparison of different cross-modal attention mechanisms.

Attention	Parameter (%)			
	IoU	PA	Precision	F1
MCANet	92.37	95.68	96.15	96.04
DDFNet	92.89	96.55	96.05	96.23
CIF	93.28	96.77	96.37	96.52

**Table 4:** Learned weight parameters in the dynamic fusion modules.

Fusion Stage	α (RGB Weight)	β (MS Weight)	Ratio (α:β)
Feat4	1.107	0.971	1.14:1
Feat5	0.892	0.915	0.97:1

**Table 5:** Ablation study on the AED.

AED	Parameter (%)			
	IoU	PA	Precision	F1
w/o CBAM	93.28	96.77	96.37	96.52
w/CBAM	93.42	96.82	96.38	96.60

**Table 6:** Performance comparison with state-of-the-art cross-modal models.

Model	Parameter (%)			
	IoU	PA	Precision	F1
BiSRNet	88.98	93.37	92.75	92.50
RTFNet	91.13	94.88	95.09	95.39
DFAFNet	81.23	90.45	91.76	89.65
DCA-UNet	93.42	96.82	96.38	96.60

**Table 7:** Influence of different input modalities on DCA-UNet performance.

Model	Input		Parameter (%)			
	RGB	MS	IoU	PA	Precision	F1
DCA-UNet	√ ^1^		87.67	95.41	91.53	93.43
		√	87.41	91.03	85.14	87.99
	√	√	93.42	96.82	96.38	96.60

^1^ The checkmark (√) in the table indicates the selected input data for the model.

**Table 8:** Generalization performance of DCA-UNet across different flight altitudes.

Height			Parameter (%)			
60 m	100 m	150 m	IoU	PA	Precision	F1
√ ^1^			90.59	94.06	96.09	95.06
	√		93.42	96.82	96.38	96.60
		√	89.22	94.22	94.38	94.30
√		√	89.43	93.96	94.88	94.42

^1^ The checkmark (√) indicates the selected flight altitude used for testing the model’s stability with images from different heights.

## Data Availability

The data presented in this study is available on request from the corresponding author. The data is not publicly available due to privacy.

## Abbreviations

The following abbreviations are used in this manuscript:

DCA-UNet	Dual-branch Cross-modal Attention-enhanced UNet
DBE	Dual-branch Encoder
CIF	Cross-modal Interaction Fusion module
AED	Attention-enhanced Decoder
CotSR	Cross-modal Spatial Reasoning
CBAM	Convolutional block attention module
MLP	Multi-Layer Perceptron
IoU	Intersection over Union
PA	Pixel Accuracy
